# Transcriptome Analysis of lncRNA and mRNA Expression Profiles During Safflower (*Carthamus tinctorius*) Seed Germination and Seedling Establishment

**DOI:** 10.3390/genes17070753

**Published:** 2026-06-30

**Authors:** Kehui Zhang, Shuo Liu, Kang Ma, Tiange Yang, Hong Liu, Lu Lv, Rui Qin

**Affiliations:** 1 Hubei Provincial Key Laboratory for Protection and Application of Special Plant Germplasm in Wuling Area of China, College of Life Sciences, South-Central Minzu University, Wuhan 430074, China; zkehui8080@163.com (K.Z.);; 2 State Key Laboratory of Plant Diversity and Specialty Crops, Wuhan Botanical Garden, Chinese Academy of Sciences, Wuhan 430074, China; 3 State Key Laboratory of Maize Bio-Breeding, National Maize Improvement Center, Frontiers Science Center for Molecular Design Breeding, College of Agronomy and Biotechnology, China Agricultural University, Beijing 100193, China; 4 Experimental Teaching and Engineering Training Center, Undergraduate School, South-Central Minzu University, Wuhan 430074, China

**Keywords:** *Carthamus tinctorius* L., transcriptome, seed germination, seedling establishment, long non-coding RNAs

## Abstract

**Background:** Safflower (*Carthamus tinctorius* L.) is a high-value economic crop with broad applications in agriculture, industry, and traditional medicine. Seed germination and seedling establishment are critical stages in the safflower life cycle, as they directly influence subsequent seedling establishment, survival, and plant growth. However, the transcriptomic dynamics and regulatory mechanisms underlying these processes remain largely unexplored, and the functional roles of long non-coding RNAs (lncRNAs) in this context are also poorly understood. **Methods:** In this study, transcriptome sequencing was performed across five developmental stages from seed germination to seedling establishment in safflower, followed by a comprehensive transcriptomic analysis and lncRNA identification. **Results:** Transcriptome sequencing identified a total of 3027 lncRNAs, including 940 natural antisense transcript (NAT)-pair-associated lncRNAs, which were classified into the divergent, convergent, and chimeric categories. Among these, 767 lncNATs were differentially expressed. Further analysis identified 542 NAT pairs in which both the protein-coding gene and its corresponding lncNAT exhibited a differential expression across the five developmental stages. A functional enrichment analysis of the predicted target genes of these lncRNAs suggested their involvement in photosynthesis and hormone-related responses. An enrichment analysis of differentially expressed genes (DEGs) across developmental stages further revealed the significant enrichment of photosynthesis and plant hormone signal transduction-related pathways, suggesting that these pathways are closely associated with safflower seed germination and seedling establishment. A further analysis of photosynthesis-related genes, particularly the expression patterns of LHC family members, suggested that Stage 3 may represent an important developmental transition associated with the optimization of the light-harvesting capacity during early seedling establishment, whereas plant-hormone-related genes are involved in regulating seed germination and subsequent leaf growth during seedling establishment. In addition, a weighted gene co-expression network analysis (WGCNA) identified candidate transcription factors associated with photomorphogenesis and plant hormone responses in safflower. **Conclusions:** This study advances our understanding of the regulatory mechanisms underlying safflower seed germination and subsequent growth and provides valuable molecular resources for future safflower breeding programs.

## 1. Introduction

*Safflower* (*Carthamus tinctorius* L.) is an annual herb belonging to the genus *Carthamus* in the Asteraceae family, which has been cultivated worldwide as a source of edible oil and dyes for hundreds of years. In addition, Safflower is suitable for planting in dry areas because of its relative drought- and salt-tolerant properties [1]; China, the United States, Mexico, Argentina, and India are the main production countries of this crop [2]. Safflower has been cultivated in China for over two millennia and is an important economic crop used as a medicinal herb, natural dye, oilseed, and animal feed [3], with its pigments widely utilized as natural colorants in food, beverages, and cosmetics [4]. In the future, safflower oil has the potential to be widely used for biofuel production [5]. Modern pharmacological studies indicate that safflower possesses significant medicinal potential, as its extracts exhibit diverse biological activities and have been used to treat menstrual disorders, cardiovascular diseases, pain, and trauma-induced swelling [6].

As the initial stage of plant growth and establishment, seed germination is closely associated with seedling establishment and the final crop yield and is regulated by multiple factors, including temperature, water, light, and plant hormones [7,8]. Studies have shown that light is a key regulatory factor in seed germination and seedling establishment, while plant hormones also play important roles in this process [9]. Under suitable temperature and moisture conditions, seeds can remain dormant. The release of dormancy is strongly influenced by hormonal changes within the seed as well as external factors such as temperature and light [10]. In species such as Arabidopsis and tobacco, light acts as a key regulatory signal that is perceived by seeds and transduced to modulate internal physiological processes during germination [11]. Furthermore, seedling establishment is also closely influenced by light. Both light perception in seeds and photomorphogenesis in seedlings play pivotal roles in seed germination and subsequent establishment. In addition, hormonal changes within seeds regulate germination through signal transduction pathways that control the expression of related genes. Studies in rice have shown that seed germination is primarily regulated by the abscisic acid (ABA) and gibberellin (GA) pathways, which act as key regulators of this process [9].

As photoautotrophs, the seedling establishment of the photosynthetic capacity is essential for seedling establishment and energy transfer from tissues to organelles. The photosynthetic system consists of Photosystem I (PSI) and Photosystem II (PSII), which convert absorbed solar energy into chemical energy, enabling plants to perform photosynthesis and maintain normal physiological functions. Within this system, PSI and PSII collaborate to facilitate the transfer of photoelectrons, catalyzing the movement of electrons from H_2_O to NADP^+^ by generating strong reducing agents and establishing a proton gradient that drives ATP synthesis [12]. The light-harvesting complex (LHC) is a complex composed of chlorophyll-binding proteins located in the reaction centers of PSI and PSII. It functions as an antenna, collecting and funneling captured light energy to the reaction centers to drive photochemical reactions; this process optimizes the efficiency of photosynthesis by ensuring effective energy transfer and utilization [13]. During seed germination, photomorphogenesis plays a crucial role in seedling growth and establishment. The expression of LHC proteins regulates key physiological and biochemical changes associated with photomorphogenesis, including the inhibition of hypocotyl elongation, cotyledon opening and greening [14], and apical hook straightening [15]. The LHC family consists of two groups, Lhca and Lhcb, which are associated with PSI and PSII, respectively [16]. In species such as algae, Arabidopsis thaliana, and tea, LHC proteins play important roles in photosynthesis [17], particularly Lhcb1 and Lhcb2 in Arabidopsis thaliana [18].

Plant hormones play central roles in regulating seed dormancy, germination, and various physiological processes. Studies in Arabidopsis thaliana have shown that the transition from seed to seedling is not solely controlled by abscisic acid (ABA), but also involves other hormones, including ethylene [19], brassinosteroids [20], cytokinins [21], and gibberellins (GA) [22]. Cytokinin promotes seed germination by downregulating Arabidopsis Response Regulators (ARRs) in the ABA signaling pathway, thereby alleviating ABA-mediated inhibition. Ethylene modulates germination by interacting with ABA and GA signaling pathways and influencing their biosynthesis through gene expression and protein regulation [23]. In addition, indole-3-acetic acid (IAA) and jasmonic acid (JA) synergistically enhance ABA signaling to maintain seed dormancy and suppress germination. The crosstalk among ABA, JA, and IAA coordinately regulates seed germination dynamics [24].

Long non-coding RNAs (lncRNAs) are functional RNA molecules longer than 200 nucleotides that play crucial roles in plant growth, development, and stress responses [25]. Accumulating evidence indicates that lncRNAs regulate gene expression at multiple levels, including chromatin remodeling, transcription, pre-mRNA splicing, and translation. In plants, certain lncRNAs also contribute to genome stability and are induced in response to abiotic and biotic stresses, such as drought, temperature fluctuations, and pathogen infection [26]. In addition, lncRNAs have been implicated in seed development and seedling establishment, thereby influencing plant growth and organogenesis across multiple developmental stages. They have also been shown to affect protein complex assembly and regulate the translation of small non-coding RNAs and micropeptides [27]. For example, in tea plants (*Camellia sinensis*), infection by the fungal pathogen Colletotrichum camelliae impairs plant growth and development. Recent studies have shown that the lncRNA *Cslnc256* acts as a molecular decoy for *CsmiR394*, thereby preventing the *CsmiR394*-mediated degradation of the sulfate transporter gene *CsSULTR2;1*. This regulatory mechanism modulates sulfate metabolism and enhances disease resistance in tea plants [28]. However, the potential roles of long non-coding RNAs (lncRNAs) during safflower seed germination and seedling establishment remain unexplored. We hypothesize that lncRNAs likely act in regulating photosynthesis- and hormone-related pathways during these developmental transitions.

Safflower holds substantial economic value across agriculture, industrial applications, and traditional medicine. Seed germination and seedling establishment critically influence subsequent plant establishment, survival rate, and overall growth performance. In this study, we employed samples representing five consecutive developmental stages, from seed germination through to plant establishment as the material for transcriptome sequencing. Based on these data, we conducted a comprehensive integrative analysis encompassing transcriptome profiling, lncRNAs identification, and weighted gene co-expression network analysis (WGCNA). This study aimed to elucidate the dynamic patterns of gene expression and regulatory mechanisms underlying the transition from seed germination to seedling establishment in safflower.

## 2. Materials and Methods

### 2.1. Plant Material and Growth Conditions

Seeds of the Anhui-1 safflower cultivar were sourced from the seed resource bank at South-Central Minzu University. Under controlled environmental conditions (10,000 lx light intensity, 24–26 °C, and a 16 h photoperiod), seeds were first soaked in water for 24 h and then transferred to vermiculite for germination. The soaked seeds were collected as Stage 1 samples. Leaves of seedlings after germination were harvested at 3, 5, 7, and 10 days post-germination (Stages 2–5), immediately frozen in liquid nitrogen, and stored at −80 °C for subsequent transcriptomic analysis. For each developmental stage, three independent biological replicates were collected. Each biological replicate represented one independent biological sample, and the corresponding tissue was sampled separately for transcriptomic analysis.

### 2.2. RNA Extraction, cDNA Library Construction, and Illumina Sequencing

Total RNA was extracted from 50 to 100 mg of tissue stored at −80 °C using Trizol reagent, following the manufacturer’s protocol (Invitrogen, Carlsbad, CA, USA). RNA concentration and purity were assessed using a NanoDrop spectrophotometer, and integrity was evaluated with an Agilent 2100 Bioanalyzer. Only samples meeting the following criteria were used for downstream analyses: total RNA ≥ 20 ng, concentration ≥ 400 ng/μL, and an OD260/280 ratio of 1.8–2.2. Qualified RNA samples were subsequently used for RNA-seq library construction. mRNA was enriched from total RNA using oligo (dT) magnetic beads, and cDNA libraries were constructed with an average insert size of approximately 350 bp (±50 bp). Paired-end sequencing was then conducted on the DNBSEQ platform according to the manufacturer’s recommended protocol (LC Bio, Hangzhou, China).

Raw reads were processed using SOAPnuke to remove low-quality reads, adapter-contaminated reads, and reads containing excessive ambiguous bases. Sequence quality was further assessed using FastQC, and the resulting clean reads were retained in FASTQ format for subsequent analyses.

### 2.3. Transcript Assembly and lncRNA Identification

Paired-end (2 × 150 bp) reads from all samples were aligned to the Anhui safflower reference genome using HISAT2 (v2.2.1) [29] Transcript assemblies were generated for each sample based on the alignment results using StringTie (v2.1.7). Individual transcript assemblies were subsequently merged into a non-redundant transcript set using the stringtie—merge function for downstream transcriptome analyses. Transcripts shorter than 200 bp were discarded from the non-redundant set. The remaining sequences were aligned to the UniProt database (https://www.uniprot.org/ (accessed on 5 November 2025)) to exclude those with potential protein-coding capacity. Non-coding transcripts were further filtered and long non-coding RNAs (lncRNAs) were identified using PlantLncBoost, LncFinder-plant [30], and CPAT-plant [31]. Candidate lncRNAs were identified using three prediction tools, including PlantLncBoost, LncFinder-plant, and CPAT-plant, with their default parameters. Only transcripts that were simultaneously predicted as non-coding by all three tools and showed no significant similarity to known proteins in the UniProt database were retained as high-confidence lncRNAs for downstream analyses. Based on transcript genomic coordinates, bedtools [32] was used to identify lncRNAs located within protein-coding gene regions. LncRNAs transcribed from the opposite strand of their corresponding protein-coding genes were defined as natural antisense transcript (NAT) pairs.

### 2.4. Analysis of lncRNA and mRNA Expression

Sequencing reads from each sample were aligned to the Anhui reference genome using HISAT2 (v2.2.1). Expression levels of both protein-coding genes and lncRNAs were then quantified with StringTie (v2.1.7). Raw gene count matrices generated from the RNA-seq data were used as input for differential expression analysis. Differential expression analysis across different safflower materials during seed germination and seedling establishment was conducted using the DESeq2 package (v1.48.1) in R [33]. Raw read counts generated from the RNA-seq data were used as the input for differential expression analysis. Transcripts with a false discovery rate (FDR) ≤ 0.05 and |log2 (fold change)| ≥ 1 were identified as differentially expressed mRNAs or lncRNAs. Expression heatmaps for coding genes and lncRNAs were generated using the pheatmap (v1.0.13, https://github.com/raivokolde/pheatmap (accessed on 2 February 2026)) package in R. GO enrichment and KEGG pathway enrichment analyses of DEGs were performed using R package clusterProfiler (v 4.16.0) [34].

### 2.5. WGCNA Analysis and Gene Network Visualization

Co-expression networks were constructed using the WGCNA package (v1.73) [35] in R (v4.5.1). The soft-thresholding power was selected using the pickSoftThreshold function based on the scale-free topology criterion, and a power of 18 was chosen because it achieved a high scale-free topology fit while maintaining acceptable network connectivity (Figure S1). The minimum module size was set to 400 genes to reduce fragmentation of highly correlated gene clusters and to identify robust co-expression modules. Normalized expression values for each transcript were used as input data. Module eigengene values were calculated and used to assess associations with seed germination and seedling establishment stages. To visualize gene co-expression relationships, genes with the highest co-expression weights connected to each selected candidate gene within the corresponding module were extracted to construct the co-expression network. The co-expressed genes were further annotated to identify transcription factors, and the resulting networks were visualized using Cytoscape (v3.10.4).

## 3. Results

### 3.1. TranscriptomeAanalysis of Seed Germination and Seedling Establishment

**Figure 1:**
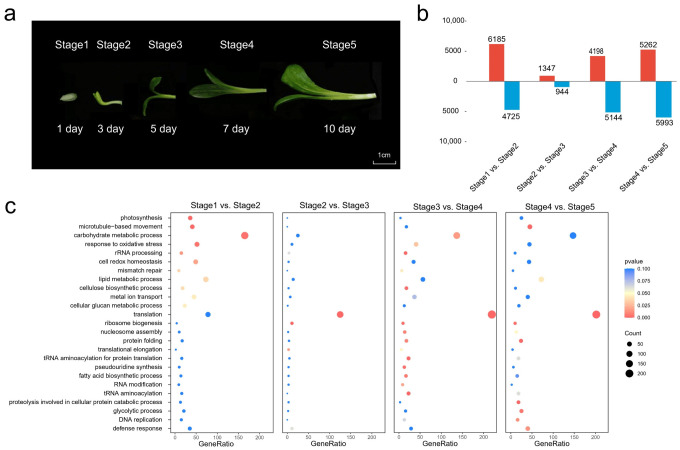
Differential expression analysis across five adjacent developmental stages during seed germination and seedling establishment in *Carthamus tinctorius* L.: (**a**) schematic of the seed germination and seedling establishment in *Carthamus tinctorius* L.; (**b**) numbers of upregulated (red bar) and downregulated (blue bar) DEGs at adjacent developmental stages; and (**c**) GO enrichment analysis of DEGs between consecutive developmental stages.

Seed germination and seedling establishment are complex, multi-step processes and represent critical stages in the plant life cycle. During germination, storage nutrients accumulated in seeds are catabolized to provide energy and substrates for seed germination and seedling establishment. This process is accompanied by substantial changes in water uptake, enzymatic activity, phytohormone regulation, cellular metabolism, and plant morphology [36]. In this study, safflower seeds were subjected to germination experiments. Seeds were first soaked in water for 24 h and designated as Stage 1 samples. Leaves were collected at 3, 5, 7, and 10 days after germination and designated as Stages 2–5, respectively (Figure 1a). At Stage 1, seeds absorbed water, the seed coat softened, and cellular metabolism was initiated and became active. During Stages 2–5, germination entered the initiation and early seedling establishment phases. The radicle emerged through the seed coat and grew downward, while the plumule elongated, penetrated the seed coat, and grew upward. Subsequently, the cotyledons expanded, and the embryo further developed into young leaves, which supported early seedling growth and initiated the production of organic compounds through photosynthesis.

A total of 15 samples representing five developmental stages, with three biological replicates per stage, were subjected to RNA-seq analysis. An average of 61.83 million reads per sample was generated (Figure S2). A differential expression analysis between adjacent stages was performed using DESeq2, with a false discovery rate (FDR) ≤ 0.05 and an absolute fold change ≥ 2.0 as the significance thresholds, to investigate the molecular mechanisms underlying safflower seed germination and seedling establishment. Principal component analysis (PCA) showed that biological replicates generally clustered together according to developmental stage, indicating the good reproducibility of the RNA-seq data while also reflecting the progressive transcriptional changes among adjacent developmental stages (Figure S3). Across the five developmental stages, different numbers of differentially expressed genes (DEGs) were identified between adjacent stages. Specifically, 10,901 DEGs were identified between Stages 1 and 2, 2291 between Stages 2 and 3, 4432 between Stages 3 and 4, and 5207 between Stages 4 and 5 (Table S1). These results indicate that a relatively large number of DEGs were identified during seed germination (Stage 1 vs. Stage 2). Following the transition to seedling establishment, the number of DEGs progressively increased with seedling growth and maturation (Figure 1b). GO and KEGG enrichment analyses revealed that DEGs were significantly enriched in photosynthesis-related pathways, particularly in the Stage 1 vs. Stage 2 comparison, suggesting that the transition from seed germination to early seedling establishment is a key phase for the initiation of photomorphogenesis and photosynthetic development. In the Stage 4 vs. Stage 5 comparison, DEGs were mainly enriched in defense-response-related pathways (Table S2). During this stage, seedling leaves displayed a narrow lanceolate morphology, and cuticular wax deposition began on the leaf surface, indicating the coordinated activation of structural and biochemical defense mechanisms associated with seedling maturation and adaptation (Figure 1a,c).

### 3.2. Analysis of Natural Antisense Transcript Pairs (NAT)

**Figure 2:**
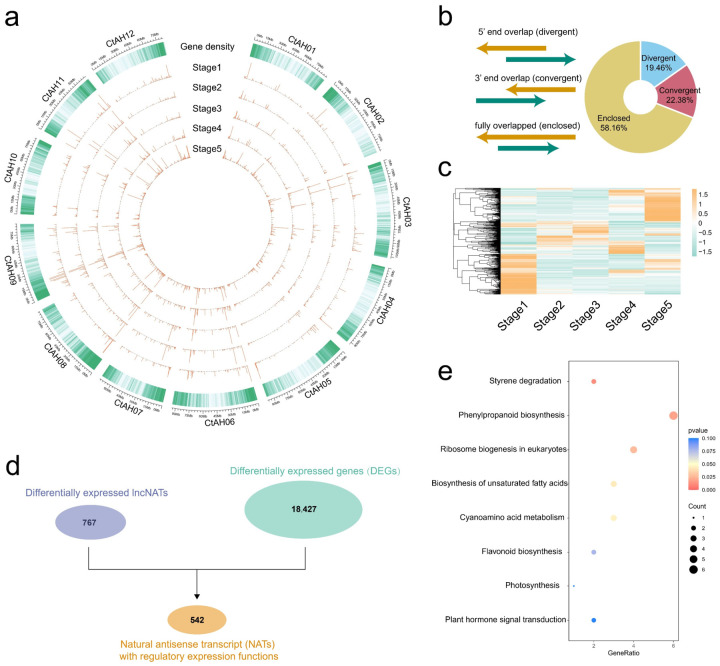
Identification of natural antisense transcript pairs (NAT) and expression patterns of lncRNAs across five developmental stages of safflower. (**a**) Distribution patterns of lncRNAs in the genome chromosome. The orange-yellow color indicates upregulation of lncRNA expression; the blue lines indicate downregulation of lncRNA expression. (**b**) Classification of NAT pairs based on the direction of transcription and the overlap region between sense and antisense transcripts. Orange arrow denotes the transcript of the coding strand, whereas the cyan arrow denotes the antisense transcripts. (**c**) Expression profiles of lncNATs across five developmental stages of safflower. (**d**) Identification of NAT pairs in which lncNATs exhibit regulatory relationships with protein-coding genes. (**e**) KEGG enrichment analysis of lncNATs that exhibit regulatory relationships with protein-coding genes.

Long non-coding RNAs (lncRNAs) participate in epigenetic and transcriptional regulation, exerting crucial roles in tissue organization, scaffolding, and the regulation of nuclear condensation. Additionally, lncRNAs can act as targets, sequester, and chaperone various protein partners to perform their functions [37]. However, studies on lncRNAs in safflower remain limited. Therefore, in this study, we systematically identified lncRNAs in safflower. A differential expression analysis of lncRNAs was performed, followed by hierarchical clustering and heatmap visualization. This analysis revealed distinct stage-specific expression patterns of lncRNAs. Specifically, cluster C1 showed preferential upregulation at Stages 2 and 3, whereas cluster C2 was specifically upregulated at Stage 5. In contrast, clusters C3 and C4 exhibited a stage-specific expression at Stage 4 and Stage 1, respectively (Figure S4). A chromosomal distribution analysis revealed that lncRNAs tended to be highly expressed in gene-dense genomic regions, indicating that they may participate in the regulation of neighboring genes or functionally associated genes (Figure 2a).

Natural antisense transcripts (NATs), a subclass of lncRNAs, are transcribed from the DNA strand opposite to protein-coding or other non-coding genes. NATs have been shown to participate in diverse regulatory pathways and regulate the expression of their cognate sense genes. By contrast, long intergenic non-coding RNAs (lincRNAs) originate from genomic regions between annotated protein-coding genes and often display tissue-, organ-, or cell-type-specific expression patterns [38]. A total of 940 lncRNA-associated natural antisense transcript (lncNAT) pairs were identified based on the overlap of genomic coordinates between lncRNAs and protein-coding genes. lncRNA–coding gene pairs showing overlapping genomic regions were considered candidate lncNAT pairs and were retained for subsequent structural classification and downstream analyses. Partial overlap was considered sufficient for defining lncNAT pairs. Based on the relative positions of the overlapping regions, the identified lncNAT pairs were classified into three structural categories: divergent, convergent, and chimeric. Divergent pairs were characterized by a 5′-end overlap, convergent pairs by a 3′-end overlap, and chimeric pairs by the complete embedding of one transcript within the other. Among these lncNAT pairs, divergent, convergent, and chimeric types accounted for 19.46%, 22.38%, and 58.16%, respectively, indicating that the chimeric configuration was the predominant structural type (Figure 2b).

A differential expression analysis showed that Stage 1 and Stage 5 contained the highest numbers of upregulated lncNATs (Figure 2c). This pattern suggests that the transitions from Stage 1 to Stage 2 and from Stage 4 to Stage 5 may be key phases during seed germination and seedling establishment. These expression changes also imply the potential involvement of lncNATs in biological processes related to seed germination, leaf expansion, and light-mediated morphogenesis. Across the five developmental stages of safflower, 18,427 DEGs and 767 differentially expressed lncNATs were identified. Further analysis identified 542 NAT pairs with a differential expression of both the protein-coding genes and their corresponding lncNATs across all five stages, indicating that these lncNATs may be involved in the regulation of gene expression (Figure 2d). A KEGG pathway enrichment analysis of the target genes associated with these 542 lncRNAs revealed significant enrichment in several pathways, including “styrene degradation”, “ribosome biogenesis in eukaryotes”, and “plant hormone signal transduction”. Among these, “styrene degradation” and “phenylpropanoid biosynthesis” were the most significantly enriched pathways. Additional enriched pathways included “flavonoid biosynthesis” and “photosynthesis”, suggesting that these lncRNAs may be involved in secondary metabolism, photosynthetic activity, and hormone-mediated regulatory processes (Figure 2e).

### 3.3. Analysis of Photomorphogenesis-Related Genes in Safflower

A KEGG enrichment analysis of DEGs revealed significant enrichment in pathways related to photosynthesis and plant hormone signal transduction. A further stage-specific KEGG enrichment analysis of DEGs between consecutive developmental stages, including Stage 1 vs. Stage 2 and Stage 3 vs. Stage 4, identified “photosynthesis–antenna proteins” and “photosynthesis” as the most significantly enriched pathways. Collectively, transcriptomic analysis suggests that plant hormone signaling and photomorphogenesis may participate in seed germination and early seedling establishment.

**Figure 3:**
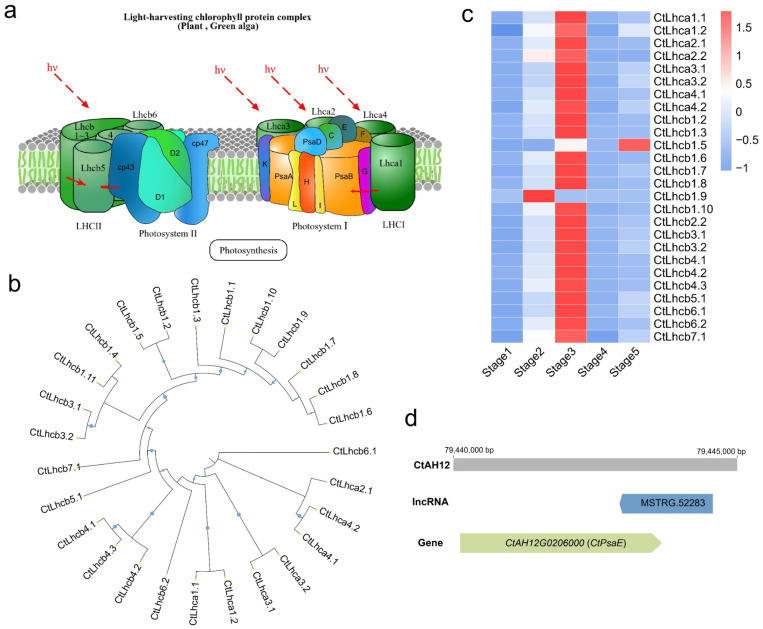
Identification and characterization of photosystem-related DEGs and lncRNAs: (**a**) photosynthetic light response pathway map in plants; (**b**) identification and phylogenetic analysis of LHC superfamily members in safflower; (**c**) expression profiles of LHC superfamily genes across five developmental stages in safflower; and (**d**) visualization of the genomic positions of lncNATs and protein-coding genes within key NAT pairs (*CtPsaE* and *MSTRG.522283*).

Light is an important factor for sustaining plant life. During the growth of plants, the process of light controlling plant development is often called photomorphogenesis. During safflower seed germination and seedling establishment, DEGs associated with photosynthesis were identified. Further analysis revealed that these genes included components related to photosystem I (PSI), photosystem II (PSII), and light-harvesting complex (LHC) proteins. LHC proteins, which are composed of light-harvesting chlorophyll a/b-binding proteins, function as antenna complexes that capture light energy and transfer it to PSI and PSII for photosynthesis. As illustrated in the photosynthetic light-reaction pathway map, Lhca and Lhcb are key components involved in light energy absorption during photosynthesis (Figure 3a).

The LHC superfamily was comprehensively identified in safflower, and 29 LHC-related genes were obtained, including 8 Lhca genes and 21 Lhcb genes (Figure 3b, Table S3). A heatmap analysis was performed based on the expression profiles of 26 LHC-related DEGs across different developmental stages. Most of these LHC genes were significantly upregulated at Stage 3, whereas *CtLhcb1.5* showed the highest expression level at Stage 5 and *CtLhcb1.9* was most highly expressed at Stage 2 (Figure 3c). These expression patterns differed from those of most other LHC genes, suggesting that *CtLhcb1.5* and *CtLhcb1.9* may play distinct roles during seed germination and early seedling establishment. At Stage 3, the seedling leaves expanded and turned green, which may enhance the photosynthetic capacity. Meanwhile, the stems became thicker, and the apical hooks gradually straightened, allowing seedlings to receive light more efficiently for photosynthesis. These results indicate that Stage 3 may represents a critical period for light absorption and photomorphogenesis in safflower seedlings. Adequate light conditions during Stage 3 may contribute to normal seedling establishment, whereas insufficient light may delay seedling growth (Figure 3c). Furthermore, our analysis showed that *CtPsaE*, a photosystem I component involved in the photosynthetic pathway, was potentially regulated by lncRNA and formed a convergent natural antisense transcript pair (Figure 3d).

### 3.4. Analysis of Plant-Hormone-Related Genes in Safflower

An enrichment analysis of DEGs between safflower developmental stages revealed the significant enrichment of the “plant hormone signal transduction” pathway (ko04075). These results suggest that plant hormones may play roles in seed germination and early seedling growth. A total of 230 DEGs were significantly enriched in the “plant hormone signal transduction” pathway (ko04075) during seed germination and seedling establishment. These DEGs were further classified into distinct hormone signaling pathways, including 39 genes involved in the abscisic acid (ABA) pathway, 79 in the auxin (AUX) pathway, 10 in the jasmonic acid (JA) pathway, 31 in the brassinosteroid (BR) pathway, 18 in the cytokinin (CTK) pathway, 19 in the ethylene (ETH) pathway, 15 in the gibberellin (GA) pathway, and 19 in the salicylic acid (SA) pathway (Table S4).

**Figure 4:**
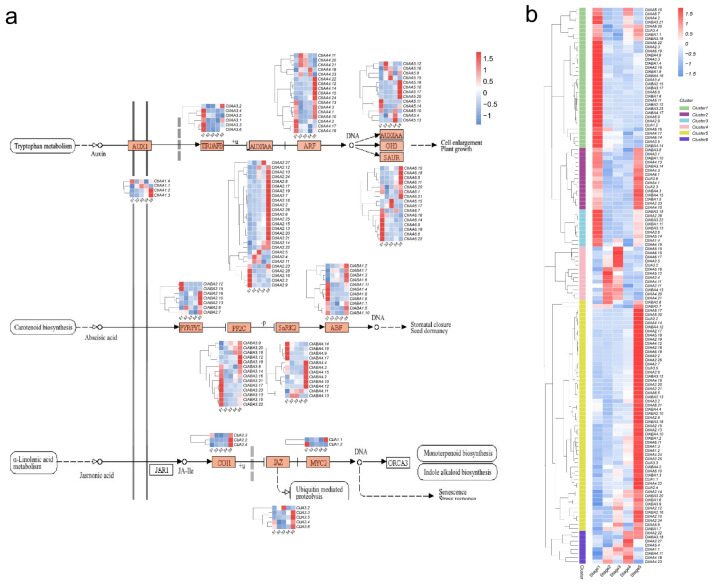
Analysis of DEGs involved in the plant hormone signal transduction pathway. (**a**) DEG expression involved in auxin, ABA, and JA signal pathways; expression levels are indicated by the heatmap for different groups. Expression levels are indicated by the heatmap for different plant hormone signal transduction-related genes. (**b**) Heatmap and clustering analysis of DEG expression in the auxin, abscisic acid (ABA), and jasmonic acid (JA) signaling pathways across five developmental stages.

ABA is widely recognized as a major plant hormone that plays a central role in controlling seed germination and early seedling growth, whereas AUX and JA coordinate with ABA signaling to reinforce the inhibition of seed germination [24]. In Arabidopsis thaliana, ABA suppresses both seed germination and early post-germinative growth, and exogenous auxin acts synergistically with JA to exacerbate ABA-mediated germination delay [39]. Specifically, during seed germination, decreased ABA levels reduce the formation of PYRABACTIN RESISTANCE/PYRABACTIN RESISTANCE-LIKE (PYR/PYL)–PROTEIN PHOSPHATASE 2C (PP2C) complexes, thereby attenuating SnRK2-mediated ABA signaling and relieving the ABA-dependent repression of seed germination [40]. In the ABA signaling pathway of safflower, seven PYR genes, fourteen PP2C genes, twelve SnRK2 genes, and eleven ABF genes were identified (Table S5). An analysis of their dynamic expression patterns showed that most genes in these four ABA-related gene families were differentially upregulated at Stages 1 and 5 (Figure 4a). Notably, during the transition from Stage 1 to Stage 2, corresponding to the seed germination phase, most ABA signaling pathway-related genes were significantly downregulated. A transcriptomic analysis suggests that ABA signaling was attenuated during germination, which may contribute to safflower seed germination.

In the AUX signaling pathway, the disruption or downregulation of TRANSPORT INHIBITOR RESPONSE 1/AUXIN SIGNALING F-BOX (TIR1/AFB) receptors or downstream AUXIN RESPONSE FACTORs (ARFs) markedly attenuates ABA signaling and alleviates seed dormancy, thereby facilitating seed germination [41]. A total of six TIR1 genes and seventeen ARF genes were differentially expressed during safflower seed germination and early seedling establishment. Among the six TIR1 genes, five were significantly downregulated during the seed germination phase, corresponding to the transition from Stage 1 to Stage 2. Similarly, seven ARF genes also showed significant downregulation during this period. These expression patterns indicate that the downregulation of TIR1 and ARF genes may attenuate the AUX-mediated regulation of ABA signaling and seed dormancy, thereby facilitating seed germination in safflower. Furthermore, in the JA signaling pathway, the upregulation of JASMONATE ZIM-DOMAIN (JAZ) genes promotes plant growth by attenuating the JA-mediated growth inhibition [42]. In safflower, most JAZ genes were significantly upregulated at Stage 5, suggesting that an enhanced JAZ expression may contribute to accelerated seedling growth and larger leaf development at this stage (Figure 4a).

Subsequently, all DEGs associated with the AUX, ABA, and JA pathways were subjected to clustering analysis based on their dynamic expression patterns, resulting in six distinct clusters (Table S6). Genes in Cluster 1 were specifically upregulated at Stage 1, and genes in Cluster 2 were specifically upregulated at both Stages 1 and 5, whereas genes in Cluster 5 were specifically upregulated at Stage 5 (Figure 4b). These expression patterns suggest that genes in Cluster 1 may be associated with seed germination, genes in Cluster 2 may participate in both seed germination and seedling establishment, and genes in Cluster 5 are mainly related to seedling growth and development. Moreover, Clusters 1, 2, and 5 each contained genes associated with the AUX, ABA, and JA pathways, suggesting that genes from these three hormone signaling pathways may act coordinately to regulate safflower seed germination and seedling growth (Figure 4b). Collectively, these transcriptomic analyses indicate that plant hormones may play crucial regulatory roles during safflower seed germination and seedling establishment.

### 3.5. Co-Expression Network Analysis with WGCNA

**Figure 5:**
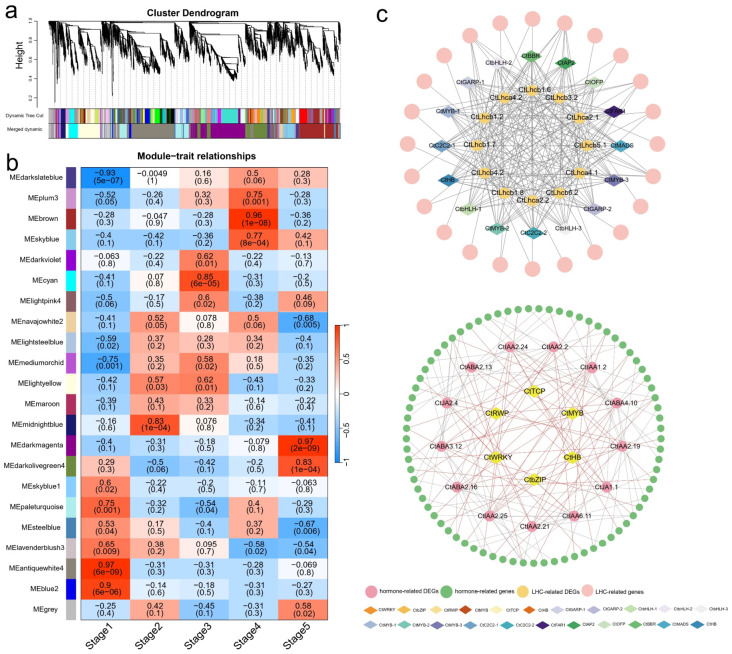
Weighted gene co-expression network analysis (WGCNA) of genes during the seed germination and seedling establishment of *Carthamus tinctorius* L. and the regulatory network of key phytohormones and photosynthesis. (**a**) Dendrogram showing the co-expression modules identified by WGCNA across seed germination and post-germination. The major tree branches constitute 22 modules labelled with different colors. (**b**) Module-stage association (Each row corresponds to a module, and each column represents a specific stage. The color of each cell at the row-column intersection indicates the correlation coefficient between a module and the stage). Red indicates a positive correlation between the module and the stage, and blue indicates a negative correlation. (**c**) Regulatory network of key phytohormones and photosynthesis. Pink circles denote DEGs associated with plant hormone metabolism, the green circles represent structural genes involved in plant hormone metabolism, the yellow circles indicate DEGs related to photosynthesis, and the light pink circles signify DEGs associated with photosynthesis. Additionally, rhombuses of distinct colors correspond to various transcription factor families identified within the same module. The transcriptional products of these transcription factors are implicated in the regulation of structural gene expression.

A co-expression network comprising all DEGs (12,593 genes) was constructed using weighted gene co-expression network analysis (WGCNA) to identify additional candidate genes potentially involved in safflower seed germination and post-germinative development (Figure 5a). For WGCNA, the soft-thresholding power was selected using the pickSoftThreshold function in the WGCNA package. A power of 18 was chosen based on the scale-free topology fit index (R^2^ > 0.9) and mean connectivity analysis (Figure S1). The minimum module size was set to 400 genes to obtain robust co-expression modules. Based on the co-expression network, 22 distinct modules were identified, and stage-associated modules were determined by correlating module eigengenes with each developmental stage (Figure 5b). Among the stage-associated modules showing correlations with developmental stages (Pearson correlation coefficient, PCC ≥ 0.80), the antiquewhite4 and blue2 modules were correlated with Stage 1, whereas the midnightblue, cyan, and brown modules were associated with Stages 2, 3, and 4, respectively. In addition, two modules, darkmagenta and darkolivegreen4, were correlated with Stage 5 (Figure 5b). To further investigate the biological processes and pathways associated with stage-specific modules, GO and KEGG enrichment analyses were performed on the combined gene sets from modules associated with each developmental stage. The results revealed distinct enrichment patterns during seed germination and post-germinative development. At Stage 1, the KEGG pathway “plant hormone signal transduction” was significantly enriched (Figure S5). At Stage 3, several KEGG pathways, including “carbon metabolism” and “nitrogen metabolism”, were enriched, while the GO term “photosynthesis” showed the enrichment (Figures S6 and S7). At Stage 5, the KEGG pathways “phenylpropanoid biosynthesis” and “plant hormone signal transduction” were significantly enriched (Figure S8).

Stages 3 and 5 represent critical periods for photomorphogenesis and rapid leaf growth in safflower seedlings, respectively. Accordingly, the cyan and darkmagenta modules showed the strongest correlations with Stages 3 and 5, respectively. Therefore, these two modules were selected for further analysis to identify candidate regulatory genes involved in safflower seedling photomorphogenesis and leaf growth. Within the cyan module, several highly connected transcription factors (TFs), including MYB, bHLH, AP2, and FAR-RED IMPAIRED RESPONSE 1 (FAR1), were identified as key regulators of the photosynthetic response. Among them, MYB TFs are known to regulate chloroplast biogenesis and the expression of photosynthesis-related genes [43], whereas FAR1 mediates light signal transduction, thereby contributing to photosynthetic regulation (Figure 5c) [44]. The darkmagenta module exhibited the strongest correlation with Stage 5. Based on its highly connected transcription factors (TFs) and KEGG enrichment analysis, this module is primarily associated with plant hormone signal transduction pathways (Figure S8). For instance, basic leucine zipper (bZIP and *CtbZIP*) TFs modulate plant responses to biotic stresses, such as pest and pathogen attacks, through hormonal signaling [45], while WRKY TFs (*CtWRKY*) interact with other TFs and contribute to the regulation of hormone biosynthesis (Figure 5c, Table S7) [46].

## 4. Discussion

*Carthamus tinctorius* L., an annual herb of the Compositae family, is a rapidly emerging oil crop of increasing significance both domestically and internationally and is widely cultivated worldwide. Its flowers have medicinal value, playing a crucial role in the treatment of cardiovascular diseases, while its seeds can be processed to produce high-quality edible oil [47]. The germination and development of safflower seeds are influenced by multiple environmental and biological factors. Moreover, traditional cultivation and harvesting practices are highly susceptible to weather conditions, often resulting in low yields and high labor costs. Therefore, elucidating the mechanisms governing safflower seed germination and seedling establishment is of substantial importance for improving crop productivity and quality.

Transcriptomic analyses were conducted to investigate RNA-level changes during safflower seed germination and seedling establishment. A differential expression analysis revealed that genes exhibiting significant expression changes (DEGs) were predominantly enriched in pathways associated with plant hormone signal transduction and photosynthesis. Long non-coding RNAs (lncRNAs) were systematically identified across the five developmental stages using an integrative pipeline combining UniProt-based protein-coding sequence filtering with three complementary coding-potential prediction tools, resulting in the identification of 3027 high-confidence lncRNAs. Among these, 767 differentially expressed lncNATs were identified. Further analysis identified 542 NAT pairs in which both the protein-coding gene and its corresponding lncNAT exhibited a differential expression across the five developmental stages. It should be noted that Stage 1 samples consisted of soaked seeds, whereas Stages 2–5 samples consisted of leaves collected from post-germination seedlings. Therefore, transcriptional differences observed between Stage 1 and later stages may reflect not only developmental transitions associated with seed germination and early seedling establishment but also differences in tissue type. Gene Ontology and pathway enrichment analyses indicated that these 542 lncNATs were significantly associated with biological processes and pathways such as “styrene degradation”, “ribosome biogenesis in eukaryotes”, and “plant hormone signal transduction”. These results suggest that phytohormones and other regulatory molecules may play pivotal roles in safflower seed germination and growth, warranting further functional investigation.

The complex formed by the binding of light-harvesting complex (LHC) proteins to pigment molecules is essential for photosynthesis, capturing and transferring solar energy. The LHC superfamily, a plant-specific group, includes light-harvesting chlorophyll a/b-binding proteins (Lhc), light-harvesting-like proteins (Lil), photosystem II subunit S (PsbS), and ferrochelatase II (FCII) [48]. Beyond light energy capture, LHC complexes play pivotal roles in regulating plant stress responses [49] and modulating growth and development [50]. In this study, 29 LHC genes were identified as differentially expressed genes (DEGs), including 8 Lhca and 21 Lhcb genes, displaying distinct expression patterns across developmental stages. A cluster heatmap analysis showed that, except for *CtLhcb1.5* and *CtLhcb1.9*, most *CtLHCs* were highly expressed at Stage 3. Interestingly, *CtLhcb1.5* was predominantly expressed at Stage 5, while *CtLhcb1.9* peaked at Stage 2. These results indicate that Stage 3 is a critical period for light energy capture during seed germination, with most *CtLHCs* highly expressed to facilitate photomorphogenesis. Overall, the expression of antenna protein DEGs is tightly and dynamically regulated throughout seed germination and seedling establishment.

Seed germination and seedling establishment are regulated not by a single hormone, but through the interactive effects of multiple hormones that collectively influence developmental outcomes. According to the hormone balance theory, the dynamic interplay and mutual inhibition among different hormonal activities determine whether germination proceeds [48]. In this study, ABA, AUX, and JA were found to interact and exert either stimulatory or inhibitory effects on safflower seed germination. During seed germination and seedling establishment, DEGs associated with ABA were highly expressed in both Stage 1 and Stage 5. For instance, *CtABF.8*, *CtPYR.12*, and *CtSnRK2.9* were predominantly expressed at Stage 1, while *CtPYR.10*, *CtPP2C.12*, and *CtSnRK2.5* showed an elevated expression at Stage 5. Moreover, *CtPP2C.14* and *CtPP2C.15* were highly expressed in both stages. These results suggest that ABA accumulation in the early stages may facilitate subsequent seed maturation, and an optimal ABA concentration can support seedling growth [51]. AUX and JA further modulate ABA activity; during early germination, they inhibit ABA via the complex formation or degradation of regulatory factors, promoting germination in the AUX signaling pathway [52]. The disruption or downregulation of TIR1/AFB receptors or downstream ARFs markedly attenuates ABA signaling and alleviates seed dormancy, thereby facilitating seed germination. In the seedling stage, AUX-related genes such as *CtAUX.10*, *CtAUX.17*, and *CtSAUR.10* exhibit a significantly increased expression at Stage 5, contributing to the promotion of seedling establishment. Furthermore, in the JA signaling pathway, the upregulation of JAZ genes such as *CtJA3.3*, *CtJA3.5,* and *CtJA3.6* may promote plant growth by attenuating JA-mediated growth inhibition.

In WGCNA, the cyan module showed the strongest association with axillary shoot elongation and cotyledon expansion at the third developmental stage. This module is significantly enriched in genes related to photosynthesis and metabolic processes, highlighting the crucial role of LHC during seed germination. The darkmagenta module contains genes positively correlated with seedling establishment, particularly at Stage 5. A functional enrichment analysis indicated the significant overrepresentation of pathways such as phenylpropanoid biosynthesis and plant hormone signal transduction, underscoring the importance of hormonal regulation in seedling growth. Notably, plant hormone signal transduction is also enriched in other modules, including lightyellow, which is highly expressed at Stages 2 and 3. Overall, these results suggest that photosynthesis and plant hormone signaling may contribute to seed germination and seedling establishment.

## 5. Conclusions

In this study, transcriptomic analyses were performed to elucidate the dynamic gene expression patterns underlying safflower seed germination and subsequent seedling development. Moreover, a total of 3027 lncRNAs were identified, including 940 natural antisense transcript (NAT)-associated lncRNAs. Among these, 767 lncNATs were differentially expressed, and 542 exhibited a differential expression across all five developmental stages. A KEGG pathway enrichment analysis indicated that lncRNAs were significantly enriched in plant hormone signal transduction pathways, suggesting that they may play regulatory roles in safflower development. A functional enrichment analysis of DEGs across successive developmental stages of safflower from seed germination revealed significant enrichment in plant hormone signal transduction and photosynthesis pathways. A further analysis of genes related to photosynthesis and plant hormone signaling indicated that Stage 3 represents a critical developmental stage for photomorphogenesis in safflower seedlings, whereas Stage 5 corresponds to a key stage for leaf growth. Additionally, genes involved in plant hormone signal transduction were found to participate in seed germination and may exert regulatory effects. Furthermore, WGCNA was employed to identify candidate transcription factors associated with photomorphogenesis and seedling growth. Overall, this study offers a systematic insight into the hormonal and photomorphogenic regulatory mechanisms governing safflower seed germination and seedling establishment.

## Data Availability

The raw sequence data reported in this paper have been deposited in the Genome Sequence Archive (Genomics, Proteomics & Bioinformatics 2025), in the National Genomics Data Center (Nucleic Acids Res 2026), and the China National Center for Bioinformation/Beijing Institute of Genomics, Chinese Academy of Sciences (GSA: CRA039439), which are publicly accessible at https://ngdc.cncb.ac.cn/gsa (accessed on 5 November 2025).

## Supplementary Materials

The following supporting information can be downloaded at: https://www.mdpi.com/article/10.3390/genes17070753/s1, Figure S1: Expression patterns of LncRNAs across five developmental stages of Safflower; Figure S2: KEGG enrichment in the antiquewhite4 module; Figure S3: KEGG enrichment in the cyan module; Figure S4: Go enrichment in the cyan module; Figure S5: KEGG enrichment in the darkmagenta module; Figure S6: KEGG enrichment in the cyan module; Figure S7: Go enrichment in the cyan module; Figure S8: KEGG enrichment in the darkmagenta module; Table S1: Differentially expressed genes in *Carthamus tinctorius* L. among Stage 1 vs. Stage 2, Stage 2 vs. Stage 3, Stage 3 vs. Stage 4 and Stage 4 vs. Stage 5.; Table S2: Significantly enrichment KEGG and GO(bp) terms associated with differentially expressed genes in *Carthamus tinctorius* L. between stages 1 and 2, stages 2 and 3, stages 3 and 4, and stages 4 and 5; Table S3: The genes involved in LHC protein and their expansion; Table S4: The genes in “plant hormone signal transduction” pathway; Table S5: The genes in “plant hormone signal transduction” pathway; Table S6: DEGs enriched in “plant hormone signal transduction”; Table S7: Annotation of transcription factors (TFs) associateed withplant hormone and LHC protein.
