# Supplementary material for: Transcriptome Analysis of lncRNA and mRNA Expression Profiles During Safflower (Carthamus tinctorius) Seed Germination and Seedling Establishment
Source: Genes (Basel). 2026 Jun 30;17(7):753. doi: 10.3390/genes17070753 (PMC13408914; doi:10.3390/genes17070753)
Supplement: Supplementary file 1 [file genes-17-00753-s001.zip › Supplementary Figure.pdf]

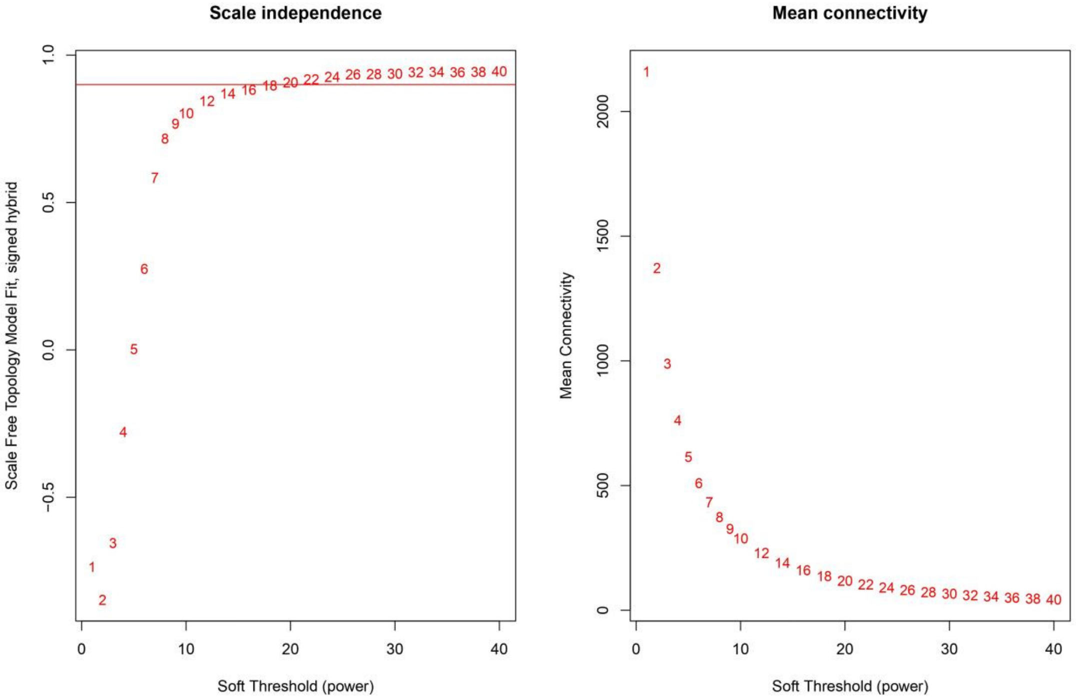

Figure S1 Determination of the soft-thresholding power for weighted gene co-expression network analysis (WGCNA). The left panel shows the scale-free topology fit index (signed R^2) as a function of the soft-thresholding power (β), and the right panel shows the corresponding mean connectivity. A soft-thresholding power of 18 was selected to construct the co-expression network.

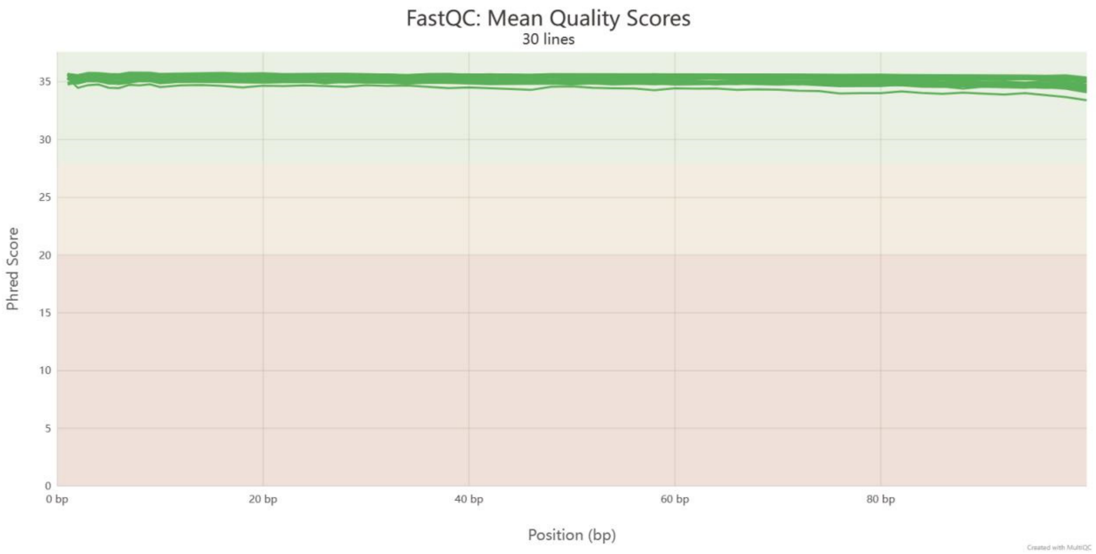

Figure S2 Sequencing quality assessment of RNA-seq data.The per-base sequence quality of all RNA-seq libraries was evaluated using FastQC. The mean quality scores across sequencing reads were consistently high, indicating good sequencing quality and suitability for subsequent transcriptomic analyses.

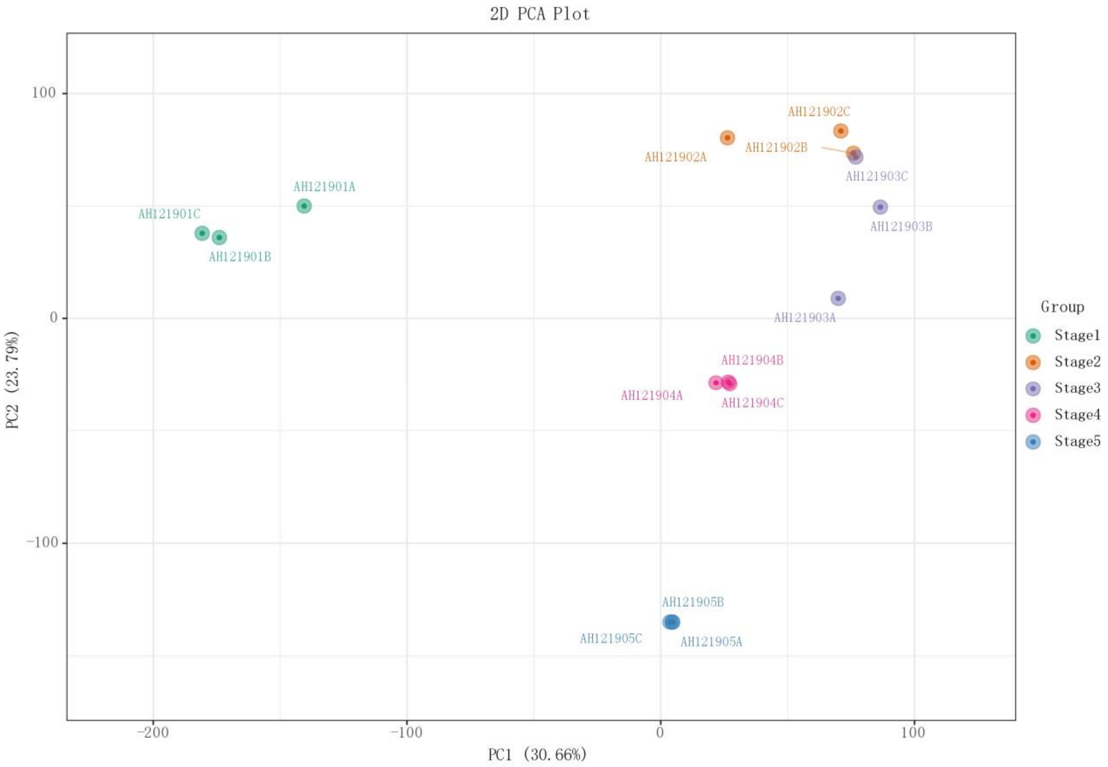

Figure S3 Principal component analysis (PCA) of RNA-seq samples from five developmental stages. PCA-derived score plots of PC1 (30.66%) and PC2 (23.79%) are shown. Each point represents one biological replicate (n = 3 per stage).

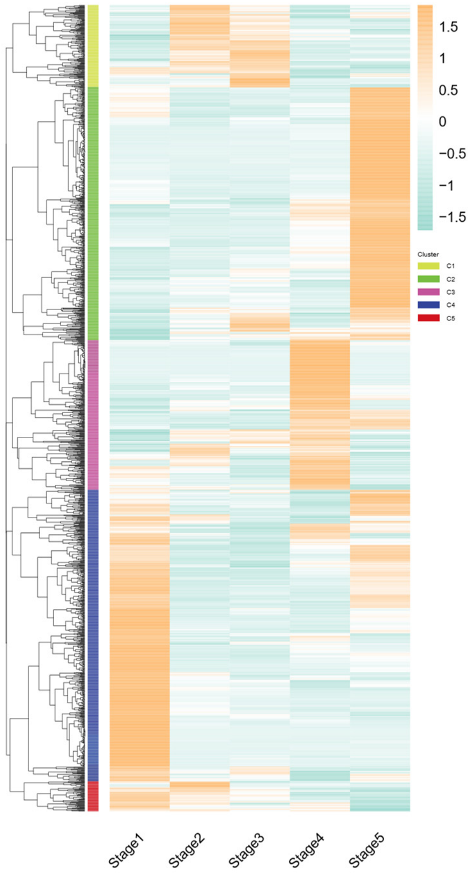

Figure S4 Expression patterns of LncRNAs across five developmental stages of Safflower

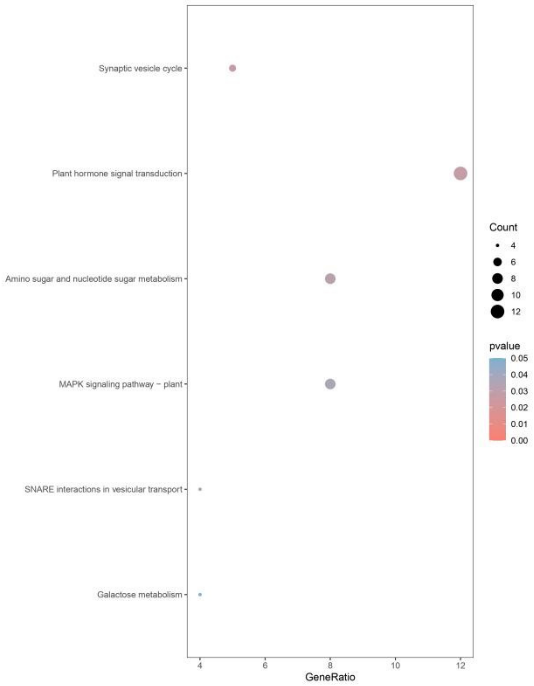

Figure S5 KEGG enrichment in the antiquewhite4 module

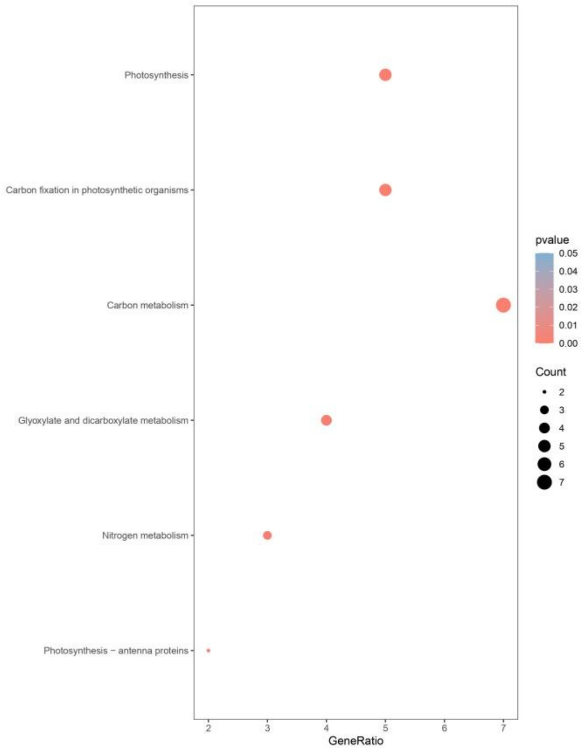

Figure S6 KEGG enrichment in the cyan module

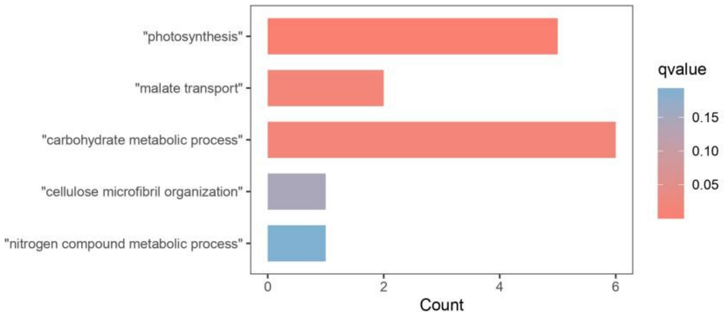

Figure S7 Go enrichment in the cyan module

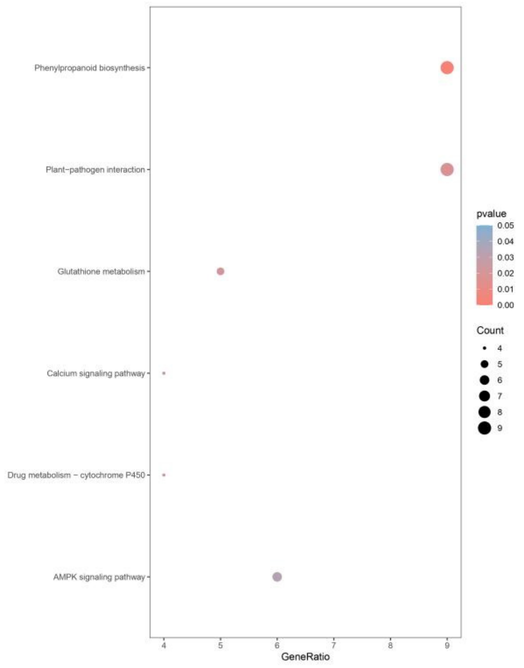

Figure S8 KEGG enrichment in the darkmagenta module
